# Growth mechanism and microstructures of Cu_2_O/PVP spherulites†

**DOI:** 10.1039/d2ra03302j

**Published:** 2022-07-11

**Authors:** Weihao Sun, Wuzong Zhou

**Affiliations:** School of Chemistry, University of St Andrews St Andrews, Fife KY16 9ST UK wzhou@st-andrews.ac.uk

## Abstract

Cu_2_O spherulites are solvothermaly fabricated by using Cu(NO_3_)_2_ as the starting material and polyvinylpyrrolidone (PVP) as a multifunctional growth agent. The specimens at different growth stages are investigated by using X-ray diffraction, electron microscopy, energy dispersive X-ray spectroscopy, soft X-ray emission spectroscopy and infrared spectroscopy. The formation mechanism of Cu_2_O spherulites is proposed accordingly. Hierarchically, the spherulites are composed of needle-like submicron-rods lying along the radial orientations. The submicron-rods are constructed by piling up of small Cu_2_O/PVP spheres. The embedded Cu_2_O nanocrystallites can generate a dipolar field in each along the [100] direction. They deposit at the surface of a negatively charged PVP-containing spherical core, and self-oriented along the radial directions. Therefore, all the Cu_2_O nanocrystallites would have their positively charged (100) facet facing to the core and their negatively charged (1̄00) facet turning towards to the spherulite surface, leading to a negatively charged surface of spherulites. Unlike randomly oriented nanocrystallites embedded in polymer microspheres, the spherulites would not undergo surface recrystallisation into a single crystal shell due to the restricted potential of local shift and rotation of the nanocrystallites by the Coulomb force from the core. This work provides new perspective towards the formation of spherulites and their structural properties.

## Abstract 2

Cu_2_O/PVP spherulites are synthesised using Cu(NO_3_)_2_ as precursor, PVP as a reductant/capping-agent and DMF as the solvent. The interaction between dipoles of Cu_2_O nanocrystals and a negatively charged core plays an important role in their formation.

## Graphical abstract

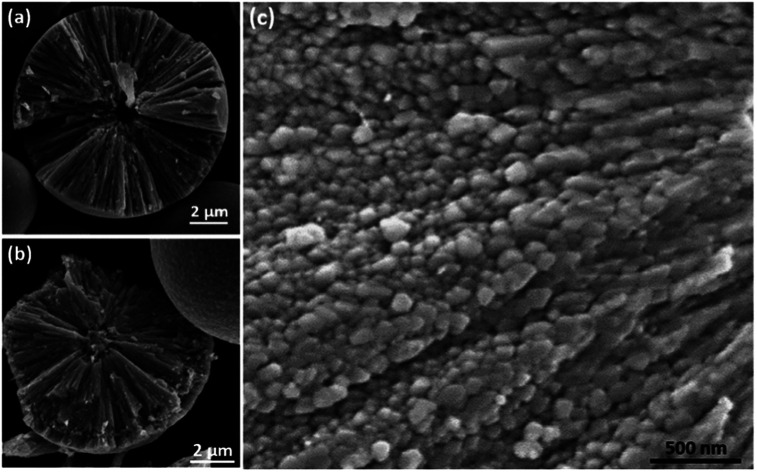

## Introduction

As discovered in early 19th century, spherulites refer to spherical aggregates of radially oriented acicular crystals.^1,2^ Spherulites are not only found in natural mineralisation,^3,4^ but can also be synthesised under biomimetic conditions with addition of polymers.^5–7^ From the energy aspect, although most spherulites seem to be homogeneous and spherically isotropic, these particles contain acicular particles as the building units, which often contain nanocrystallites embedded in a polymer substrate, leading to a high lattice energy, and making the formation hard to be explained in thermodynamics.

The formation of acicular morphology inside spherulites often associate with ordering or partial ordering of nanocrystallites. One principal argument for the driving force of self-orientation of nanocrystallites in spherulites is probably the dipolar field developed in nanocrystallites.^8–10^ Polarity is a separation of electric charge leading to an electric dipole moment in a molecule or a chemical group with negatively and positively charged ends. A bulk crystal of metal oxide normally possess no dipolar field even it is constructed alternately by negatively and positively charged atomic layers. It is because the surface roughness by horizontal zig-zag arrangement of atoms can make the surface atomic layers neutral. However, in nanocrystallites, smooth surface terminal atomic layers with either positive or negative charge can form easily, leading to a dipolar field. The formation of biomimetic vaterite spherulites is a good example,^7^ where all the nanocrystallites were self-oriented into a parallel manner when a strong electric field in the core did not exist, while turned to radial orientations when a strong dipolar field from a double layered structure was developed in the cores.

Spherulites have been found in various compositions, including naturally occurring rhyolite,^11^ corallites,^12^ and synthetic CaCO_3_,^3,5^ ZnO,^6,13^ Ni_3_Ge,^14^ polymer,^15^ MOF,^16^*etc*. Carbonate spherulites are investigated as an important geological research project in the recent years, because these millimetre sized particles may be an evidence of existence of oil-prone alginite.^17^ Their biotic growth mechanism and microstructures may carry information of the environmental conditions during their formation millions years ago.^5^ Metal oxide spherulites normally have complicated microstructures with nanocrystallites embedded in a polymer substrate. The organic substrate is a soft matter. It not only prevents further growth of the nanocrystallites, but also allow the local shift and rotation of the nanocrystallites. This property is crucial for the self-orientation of the embedded nanocrystallites in the spherulites.

In our recent research of the formation of Cu pseudo-icosahedral microcrystals using CuSO_4_·5H_2_O as precursor, polyvinylpyrrolidone (PVP) as a reductant/capping-agent and dimethylformamide (DMF) as the solvent, Cu_2_O/PVP composite spherulites were detected as an intermediate phase.^18^ The Cu_2_O nanocrystallites in these spherulites were further reduced into Cu nanocrystallites, therefore these spherulites were not preserved. Cu_2_O is a promising semiconductor and photocatalyst in reduction of CO_2_,^19^ dye degradation,^20^ H_2_ production,^21^*etc*. Various morphologies of Cu_2_O are widely studied, for instance, cube,^22^ dendrite,^23^ hollow structure,^24^ 26-facet polyhedron.^25^ However, the formation mechanism of spherulitic Cu_2_O has not been investigated in details. A research of Cu_2_O-containing in depth would not only improve our control power of Cu_2_O spherulites, but also contribute to our better understanding of general formation mechanisms of other spherulites.

Herein, we report the growth and characterisation of relatively stable Cu_2_O spherulites and proposed the formation mechanism. The effects of NO_3_^−^ anions on the reduction of copper and multifunction of PVP as an agglomerating surfactant as well as a weak reducing agent are discussed. We find that, due to alignment of nanocrystallites guided by the negatively charge PVP-rich cores, the whole surface of the spherulites is negatively charged. We anticipate this work can shed lights on the future studies in the field and control of spherulitic morphologies. Hopefully the newly obtained knowledge can be applied in crystal engineering and geology.

## Experimental

### Synthesis of Cu_2_O spherulites

The materials used in the present work were Cu(NO_3_)_2_·H_2_O (Sigma-Aldrich), PVP (Sigma, mol wt. 360 000), DMF (Acros Organics, 99+%), deionised water (DI water) with a resistivity of 18.3 M Ω cm^−1^. All chemicals were used as received without further purification.

0.5 g PVP were fully dissolved in 25 ml DMF and preheated to 150 °C before addition of 1.25 mmol Cu(NO_3_)_2_·H_2_O. The solution was stirred continuously for about 15 min when it became a green suspension. The suspension was then transferred into an autoclave vessel and put into an oven at 150 °C. The suspension was kept at this temperature for a certain time for the crystal growth. Several reaction times (1.5, 2, 6, 12, 24 and 96 h) were applied to obtain specimens from different growth stages for a study of morphology evolution and microstructural changes. When the reaction was stopped, the sample was filtered and washed three times with DI water to remove excess DMF and PVP. Finally, the obtained solid specimen was dried at 50 °C. All experiments were performed three times to make sure they were repeatable.

### Test of surface charge

To test whether the produced polycrystalline spherulites have a negatively or positively charged surface, surface stain using dyes with positive or negative charge was performed. The dyes safranin T and Congo red solutions of 0.4 gL^−1^ were firstly prepared with DI water. 0.5 g Cu_2_O spherulites with reaction time of 3 h was dispersed in 10 ml DI water to form a suspension. 10 mL suspension and 10 mL dye solution were mixed and sonicated for 5 min before staying still overnight. The suspension was then sonicated again for 5 min, followed by taking 0.5 mL onto a glass slide and being dried at 50 °C for optical microscopy observation. Parallel experiments of pure PVP were performed so as to exclude the effect of PVP's polarity towards surface stain and further prove the dipole force of Cu_2_O.

Further test to confirm the surface charge of the spherulites was agglomeration experiment. 0.05 g sodium alginate were dissolved in 10 mL DI water. The solution was stirred when 10 mL Cu_2_O suspension were added. The mixed suspension was then stirred for another 30 min before taking 10 mL to be dried at 50 °C. The solid sample was then transferred to the specimen chamber of a scanning electron microscope (SEM) for observation. In a parallel experiment, chitosan was used to replace alginate with all other conditions unchanged.

### Specimen characterisation

Powder X-ray diffraction (XRD) characterisation was performed on a STOE diffractometer in Debye–Scherrer (capillary) mode in room temperature using Cu K_α_1__ (*λ* = 1.5418 Å) radiation. The size of capillary is 0.5 mm in inner diameter. The diffracted X-rays were collected using a scintillation position-sensitive linear detector. Samples were scanned for 12 h to get their XRD patterns. SEM images were recorded using JEOL JSM-IT200 and JSM-IT800 microscopes operated from 5 kV to 15 kV. Samples were coated with a thin gold layer for 60 seconds in 10 mA to overcome the charging problem. Energy dispersive X-ray spectroscopy (EDX) was performed on the JEOL JSM-IT800 SEM operating at 15 kV. Soft X-ray emission spectroscopy (SXES) data was collected in SEM IT800 using SXES detector at −70 °C for 1 h each. Specimens for EDX and SXES studies were deposited on silica substrates, instead of carbon tapes, without gold coating to avoid detecting external carbon and gold. Fourier transform infrared spectroscopy (FTIR) data was collected on a Shimadzu IRAffinity-1s FTIR spectrometer. Transmission electron microscopy (TEM) and high resolution TEM (HRTEM) images were obtained using JEOL JEM 2011 and FEI Titan Themis 200 S/TEM (in TEM mode) both operated at 200 kV. EDX elemental mapping on FEI Titan Themis 200 was achieved in STEM mode and also at 200 kV. Samples for TEM/HRTEM observation were dispersed in acetone or ethanol, and deposited onto a TEM specimen grid coated with a holey carbon film.

## Results and discussion

**Fig. 1:**
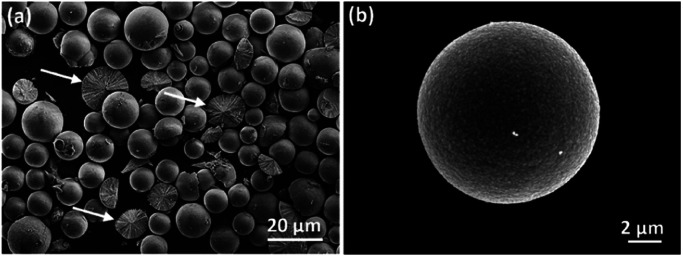
SEM images of spherulites from the samples with reaction times of (a) 1.5 h and (b) 24 h. The arrows in (a) point to some broken particles with cross section displayed.

All the samples with different reaction times from 1.5 to 96 h showed dark brown, a typical colour for Cu_2_O. SEM images revealed that the morphologies of all obtained samples were spherical with smooth surfaces as shown in Fig. 1. Some broken particles in Fig. 1a show linear components lying along the radial directions, implying that these particles are spherulites instead of common spherical particles with disordered aggregation of molecules/ions. During the process of solvothermal preparation, 1.5 h was the earliest reaction time when visible spherical solid precipitates could be collected. The majority of these spherical particles appeared to be at microscale, indicating a relatively rapid formation of the spherulites. Fig. S1 in (ESI†) shows the series of SEM images of the specimens obtained from different reaction times and the corresponding size distribution diagrams based on the measurements of 200 particles for each sample. The average size of the spherulites in 1.5 h sample is 9.5 μm in diameter. As the reaction time lengthens, the particle size increases gradually with an obvious reduction of the growth rate, *e.g*. 10.1 μm after 3 h, 10.3 μm after 6 h, 10.3 μm after 12 h, and 10.5 μm after 96 h. The increase of the particle size is not significant.

**Fig. 2:**
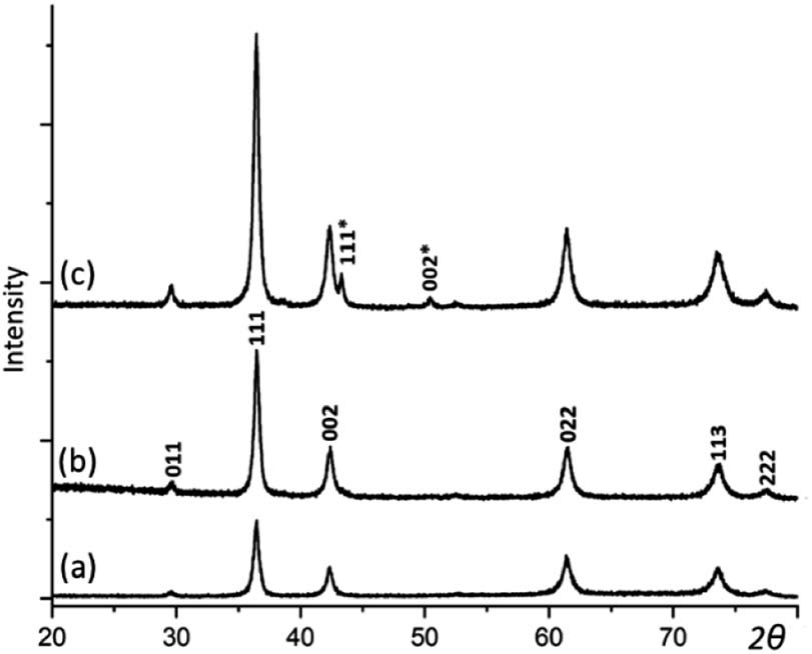
XRD patterns of the samples with the reaction time of (a) 2, (b) 6, and (c) 12 h. The pattern (b) is indexed to Cu_2_O. Two extra peaks in (c) are indexed to the Cu structure.

XRD was then applied to examine the crystalline phases in the samples and their crystallinities. As displayed in Fig. 2, when the reaction time extends, the intensity and sharpness of XRD peaks increase, indicating an improvement of crystallinity. There are two phases indicated by the series of XRD patterns chronologically. 2 h and 6 h samples appear to be pure Cu_2_O, which has a cubic structure with the unit cell parameter *a* = 4.268 Å. In 12 h sample, two extra weak peaks appear and can be indexed to the face-centred cubic structure of Cu with *a* = 3.615 Å. Cu as a minor phase is believed to be resulted from further reduction of Cu_2_O by the weak reducing agent PVP. But, this process is very slow.

The synthesis conditions of this work were almost identical to the conditions used for growth of Cu icosahedra,^18^ except in the present work the precursor CuSO_4_·5H_2_O was replaced by Cu(NO_3_)_2_·H_2_O. In the system containing sulfate, the first crystalline phase was monoclinic Cu_4_SO_4_(OH)_6_·H_2_O as observed in 1.5 h sample. These crystals were gradually reduced to form Cu_2_O nanocrystallites, which underwent aggregation to form spherulites. However, the Cu_2_O crystals were further reduced quickly to Cu. For example, in 2 h sample, the ratio of Cu_2_O to Cu was almost 1 : 1, and in 12 h sample, Cu was the only crystalline phase. In the present work using copper nitrate as the precursor, on the other hand, Cu_2_O/PVP spherulites were the only product in 1.5 h to 6 h samples. The 12 h sample contained a small proportion of Cu, indicating that Cu_2_O/PVP spherulites formed quickly and were much more stable in the nitrate system. The Cu content increased gradually when the reaction time was extended. However, even in 96 h sample, the main phase was still Cu_2_O, as discuss below.

**Fig. 3:**
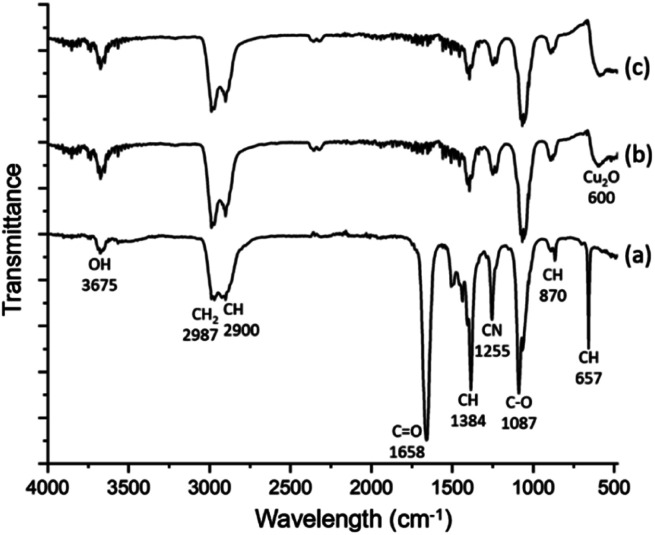
FTIR spectra of (a) DMF solution of Cu(NO_3_)_2_·H_2_O and PVP, (b) 2 h sample, (c) 6 h sample.

The spherulites are unlikely to be monophasic or single crystalline, but composed of inorganic nanocrystals and organic substrates. Spectroscopic techniques were applied to study the composition and chemical bonding in the spherulites. Fig. 3 shows FTIR spectra of the samples. The FTIR spectrum of the solution containing DMF, PVP and Cu(NO_3_)_2_·H_2_O is very similar with the spectrum of pure DMF,^26^ with additional contribution from PVP. The latter increases the peak intensities of CH_2_ (2987 cm^−1^), CH (2900, 1384 and 879 cm^−1^), and CN (1255 cm^−1^). In the FTIR spectra of 2 h and 6 h solid samples, the C=O peak (1658 cm^−1^) almost disappears, while all hydrocarbon bonds and C−O bond (1087 cm^−1^) still exist. This result indicates successful removal of the solvent DMF during extracting the precipitates, while the solid spherulites still contain PVP. The fact that the C=O peak disappears and the C–O peak remains can be attributed to the formula change of PVP when reducing Cu^2+^ and the combination of PVP with Cu cations. A similar situation was observed previously in an Ag/PVP system.^27^ Notably a new peak in these FTIR spectra at 600 cm^−1^ emerges and is assigned to crystalline Cu_2_O. Consequently, the particles are Cu_2_O/PVP composite micro-spherulites. To further confirm the involvement of PVP, SXES was performed to examine light elements in the spherulites. The spectrum is displayed in Fig. S2 in the ESI.† SXES spectrum shows the existence of C, O, N in a spherulite, therefore implies the co-existence of PVP with Cu_2_O.

**Fig. 4:**
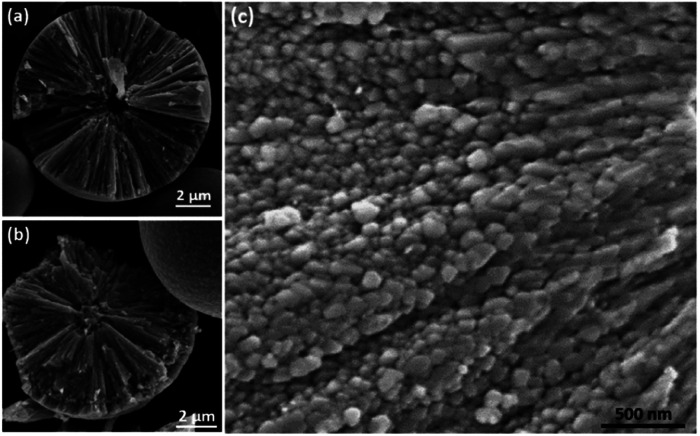
SEM images of cross sections of the Cu_2_O/PVP spherulites from broken particles. (a) Low magnification SEM image from 12 h sample, showing that the particle is constructed by needle-like components. A cavity at centre is visible. (b) SEM image from 12 h sample, showing a core at the centre of the spherulite. (c) High magnification SEM image from 1.5 h sample showing small spheres below 200 nm in diameter as building units of the needle-like components.

To reveal the inner structure of the spherulites, SEM images of cross sections of the particles were recorded as shown in Fig. 4. At a low magnification, it shows a particle in 12 h sample consisting of radially directed needle-like components with a spherical core of a diameter at about 1 μm, which was lost probably during crushing and left a cavity (Fig. 4a). Fig. 4b shows another spherulite from 12 h sample with a solid core, also about 1 μm in diameter. At a high magnification, it is revealed from 1.5 h sample that, at beginning, the needle-like components are composed by many smaller submicron particles below 200 nm in diameter (Fig. 4c). These small particles line up along the radial directions and finally merge together to form dense needle-like components as we observed in Fig. 4a and b.

**Fig. 5:**
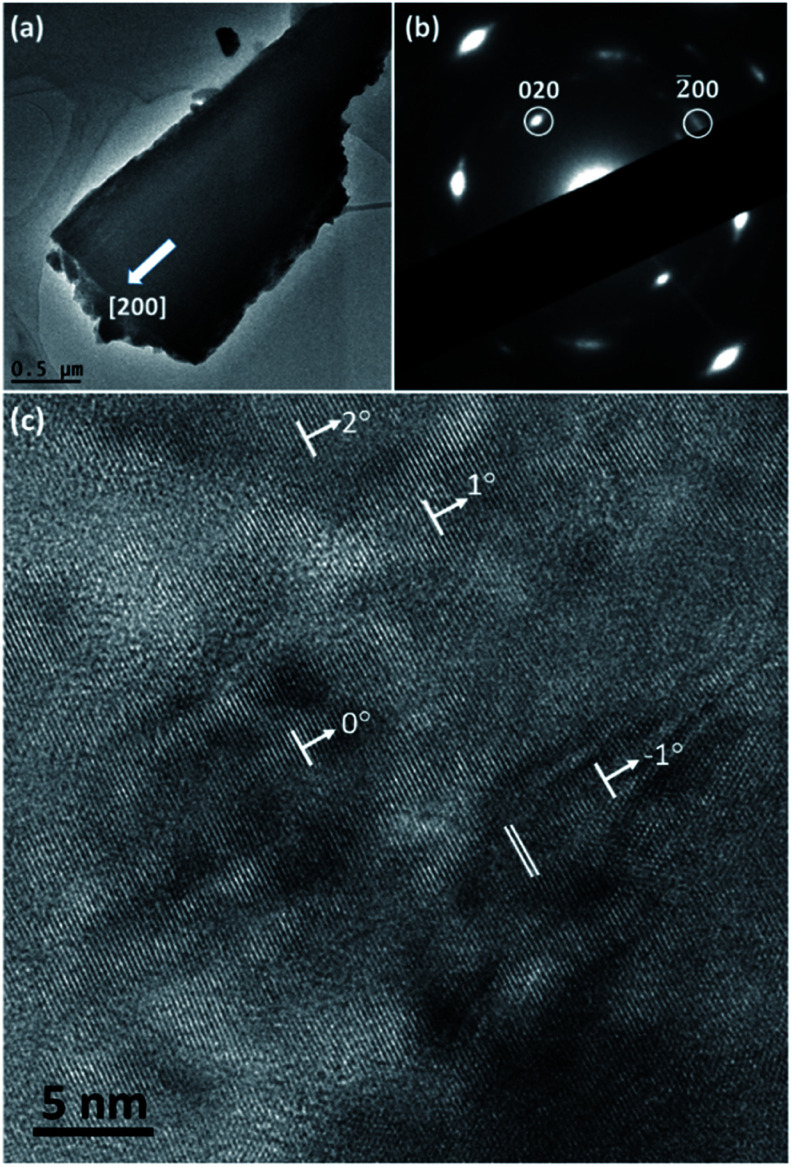
(a) TEM image of a fragment of a Cu_2_O/PVP spherulite from 1.5 h sample. The arrow indicates the radial direction in the spherulite. (b) The corresponding SAED pattern indexed to the cubic structure of Cu_2_O. (c) HRTEM image of a fragment of a spherulite from 12 h sample. The *d*-spacing of the double lines is about 2.15 Å, which can be indexed to the (200) planes of Cu_2_O. The [200] orientations of some nanocrystallites are indicated by the arrows and their angular off-alignment is marked.

TEM observation was carried out to characterise the structure of spherulites. Fig. 5a shows a TEM image of a fragment of a needle-like particle. The arrow shows the radial direction when it is in the spherulite. Fig. 5b is the corresponding SAED pattern obtained from a large area in the fragment, which can be regarded as an imperfect single crystal diffraction pattern. The principal spots can be indexed to the cubic Cu_2_O structure when viewing down the [001] direction. It is obvious that the [100] zone axis of Cu_2_O is parallel to the radial direction in the spherulite. The detailed structure of the spherulites was revealed by HRTEM images. As seen in a HRTEM image in Fig. 5c from a spherulite in 12 h sample, many nanocrystallites with fringes are visible in a disordered substrate. Their size is about 5 nm or less in diameter and their orientations are almost consistent with small angles off the alignment. The off-alignment property of the nanocrystallites leads to diffused diffraction spots as seen in Fig. 5b. The measured *d*-spacings corresponding to the fringes are about 2.15 Å, which can be indexed to the (200) planes of the cubic structure of Cu_2_O. Consequently, the produced spherulites contain self-oriented Cu_2_O nanocrystallites embedded in a PVP substrate.

**Fig. 6:**
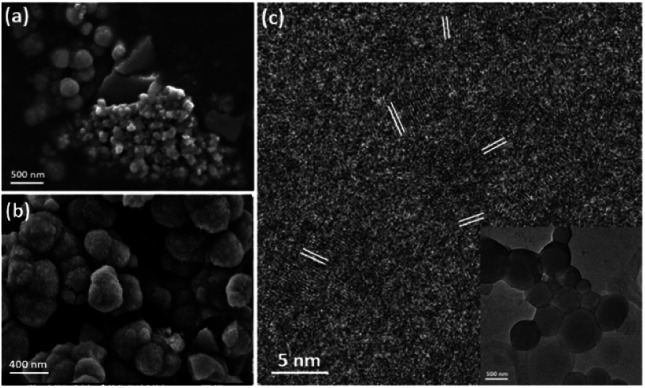
SEM images of submicron sized spheres from (a) 15 min sample and (b) 45 min sample. (c) HRTEM of a submicron sized sphere from 15 min sample showing embedded nanocrystallites with *d*-spacings 2.14 Å marked, which can be indexed to the (200) planes of cubic Cu_2_O. Inset is the low magnification TEM image of the sphere where HRTEM images were recorded.

To understand the formation mechanism of the spherulites, it is crucial to collect structural information at earlier stages. Although 1.5 h is the earliest time to collect a large amount of spherulites, formation of the submicron spherical particles which comprise the needle-like components, as shown in Fig. 4c, must take place much earlier. A DMF solution containing Cu(NO_3_)_2_ and PVP was stirred for 15, 30 and 45 min in an oil bath at 150 °C and evaporated on hotplate. Subtle greenish brown residues at these reaction times were scratched off the beaker wall and were characterised using SEM and TEM. Fig. 6a shows that the residues contain some bulk particles as well as submicron spherical particles roughly in two particle sizes, ∼300 nm and ∼60 nm in diameter. The large spherical particles with a smooth surface, already larger than the building units of the needle-like components as shown in Fig. 4c, are likely the primitive forms of the cores of the spherulites. Fig. 6b shows particles in 45 min sample. The average particle size increases to 400 nm in diameter. The surface is rough due to adsorption of many smaller particles. HRTEM images of these spheres reveal many tiny nanocrystallites with a low crystallinity embedded in a disordered substrate (Fig. 6c). The measured *d*-spacings from these nanocrystallites are about 2.14 Å, which can be indexed to the (200) planes of the Cu_2_O structure. Importantly, these nanocrystallites are randomly oriented, indicating that the interaction between the nanocrystallites is not strong enough to induce self-orientation as observed in spherulites (Fig. 5c).

Fig. S3 in the ESI† displays a TEM image and EDX elemental mapping of Cu in a submicron sphere. The distribution of Cu is quite uniform. EDX mapping of C element was not achieved because the carbon film on the TEM specimen grid could also generate strong carbon signal, interfering the mapping result. On the other hand, EDX spectrum of 15 min sample on silica substrate in SEM shows Cu, C and O, similar to the result of 1.5 h sample (Fig. S4a in the ESI†).

We presume that, when the spherical particles grew up to micrometer scale, the density of PVP increased, so did the negative charge, generating a relatively strong Coulomb electric field. Meanwhile the Cu_2_O nanocrystallites in small spherical particles also grew up to generate a dipolar field. These small spheres were attracted by the electric force and deposited on the large spheres to form spherulites very quickly. The first solid specimen we collected is 1.5 h sample, which contains spherulites with diameters of ∼10 μm.

To check the polarity of spherulites and further examine whether the driving force of the spherulite growth is dipole force, dye staining experiments and agglomeration experiments were conducted. The dyes used were safranin T (positively charged due to the amine groups) and Congo red (negatively charged due to the –SO_3_^−^ groups). Presumably, the whole particles of spherulites are neutral. If all the nanocrystallites as dipoles line up along the radial directions guided by an electric field from the negatively charged core,^28^ all the Cu_2_O dipoles would turn their positive ends towards the core and their negative ends to the surface. The whole surface of the spherulites would be therefore negatively charged and would adsorb dye molecules with an opposite charge (Fig. S5 in the ESI†).

**Fig. 7:**
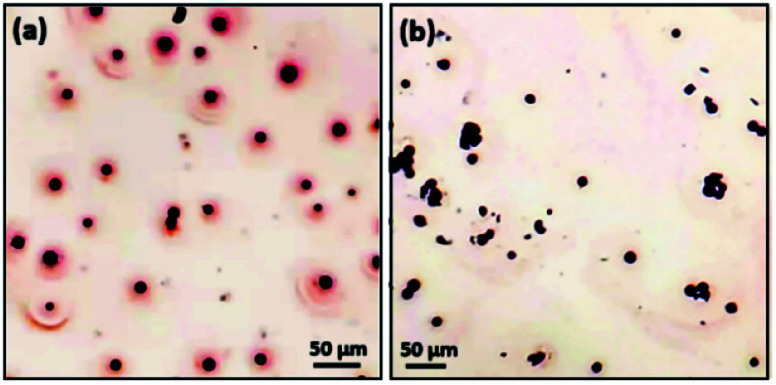
(a) Optical microscopy image of Cu_2_O/PVP spherulites with safranin T dye. The surrounding of spherulites shows blurred red, indicating the successful stain by safranin T. (b) Optical microscopy image of Cu_2_O/PVP spherulites with Congo red. The spherulites appear to be undyed, indicating the unsuccessful stain by Congo red.

In a surface stain experiment using positively charged safranin T, the produced spherulites adsorbed a lot of dye molecules to form a coating layer of about 20 μm in thickness (Fig. 7a). When safranin T was replaced by negatively charged dye Congo red, no adsorption occurred (Fig. 7b). It is noted that PVP is negatively charged. To rule out the effect of PVP's polarity during the surface dye experiments, parallel experiments of pure PVP were performed. Fig. S6(a) and S6(b) in the ESI† demonstrate a weak interaction of pure PVP with both Congo red and safranin T. PVP has no affinity with either negatively charged or positively charged dyes. Therefore, we deduce that the deposition of safranin T dye on the surface of Cu_2_O/PVP spherulites is mainly attributed to negatively charged surface due to the radially oriented dipoles of Cu_2_O nanocrystallites. It is also confirmed that the mechanism of self-orientation of the Cu_2_O nanocrystallites in the spherulites based on the Coulomb force interaction between the core and dipoles discussed above is valid.

For surfactant agglomeration experiments, Cu_2_O/PVP spherulites agglomerated on the surface of chitosan (positively charged with amine groups) in aqueous environment, while no interaction occurred with alginate (negatively charged due to acidic groups) (Fig. S6c to 6f in the ESI†). The results are consistent with the conclusions of the surface stain experiments and support our proposed formation mechanism of spherulites.

PVP plays an important role during the growth of spherulites. After reducing Cu^2+^ to Cu_2_O as a reducing agent, PVP also served as nucleation site and agglomeration agent to hold Cu_2_O nanoparticles. Furthermore, its soft matter property allows nanocrystallites to shift and rotate locally, enhancing self-orientation of the nanocrystallites.

**Fig. 8:**
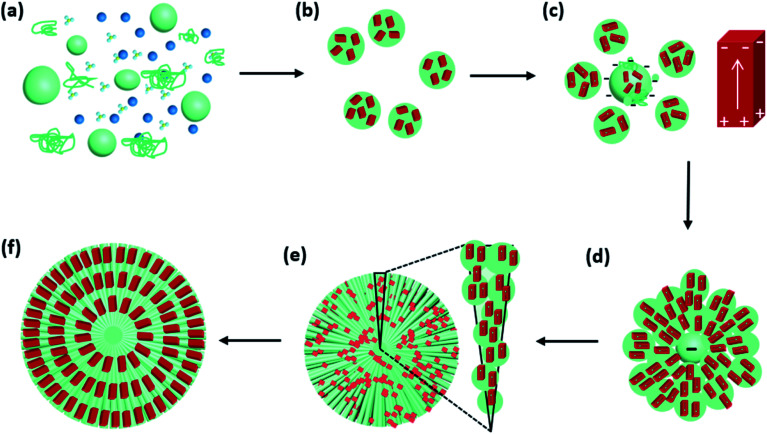
Schematic drawing illustrating the formation mechanism and morphologic evolution of Cu_2_O/PVP spherulites. (a) Co-existence of PVP cluster, Cu^2+^ and NO_3_^−^ in a DMF solution. (b) Nucleation and growth of Cu_2_O nanocrystallites in PVP/Cu_2_O submicron spheres. The Cu_2_O nanoparticles have a low crystallinity with many defects. (c) A core with deposition of small spheres, in which the Cu_2_O nanocrystallites have a high crystallinity and generate a dipolar field. The arrows point to the dipolar directions. (d) Ongoing simultaneous piling-up of PVP/Cu_2_O submicron spheres and oriental alignment of nanocrystallites. (e) Growing Cu_2_O/PVP spherulite and the zoomed-in representation of needle-like submicron rods. (f) A cross sectional structure of the final Cu_2_O/PVP spherulites.

Based on the analysis of Cu_2_O spherulites from different growth times, we now are able to propose an overall growth mechanism (Fig. 8).

Initially, Cu^2+^ cations, NO_3_^−^ ions and PVP co-exist in DMF solution at 150 °C (Fig. 8a). Commercial PVP preserves weak reducing capability.

Shortly after, Cu^2+^ cations are reduced by PVP to form Cu_2_O nanocrystallites. During this time, NO_3_^−^ anions, unlike SO_4_^2−^,^18^ do not form crystalline phase with Cu^2+^. Their decomposition can also accelerate the process of Cu^2+^ to Cu_2_O.^29^ PVP polymer serves as nanoparticle dispersant, wrapping the nanocrystallites, producing PVP/Cu_2_O microscale spheres, as witnessed in Fig. 6. The Cu_2_O nanocrystallites in these spheres are small with a low crystallinity and have random orientations. At this stage, dipolar field is not generated in the nanocrystallites (Fig. 8b). Some submicron spheres further attach PVP to grow up to large size acting as cores, which attract small spheres to their surface (Fig. 8c). The dense PVP molecules in the cores generate a strong negative centre, while the nanocrystallites in small spheres increase their crystallinity and develop a dipolar field.

The interaction between the negative core and surrounding Cu_2_O nanocrystallites leads to the self-orientation (Fig. 8d). The Cu_2_O nanocrystallites in the small spheres quickly line up along the radial directions with their positive ends facing to the core and negative ends pointing to the surface of the spherulitic particles. To achieve this, all the nanocrystallites are separated without directly merging as often observed in attachment growth of nanomaterials,^30,31^ and are able to sift and rotate locally in the PVP soft matter substrate. The alignment of Cu_2_O nanocrystallites lead to the formation the needle-like bundles (Fig. 8e). Finally spherulites form quickly with the whole surface negatively charged (Fig. 8f).

Additionally, PVP as a polymerized surfactant helps to hold the spherulite structure while itself as a weak reducing agent can further reduce Cu^+^ to Cu, as seen in Fig. 2. 12 h sample contains small proportion of Cu which was from the over reduction by PVP. Fig. S7 in the ESI† shows XRD pattern and SEM image of a cross section of a spherical particle in 96 h sample. The needle-like morphology of the inner structure disappears. We assume only under the situation of over reduction of all the Cu_2_O nanocrystallites to Cu nanocrystallites by PVP, will the dipolar fields in nanocrystallites vanish because Cu nanocrystallites would never develop dipolar field and the spherulite morphology disappears.

An important character of spherulites is that the nanocrystallites are manacled by the electric field in the core and are not parallel to each other with less freedom for local shift and rotation. A direct effect of this property is that a surface recrystallisation into single crystalline polyhedral shell would not take place in the spherulites, no matter whether they are naturally occurring spherulites,^3,5^ or synthetic spherulites.^5,6^ On the other hand, formation of spherical aggregates of disordered materials without charged cores is an important step in reversed crystal growth. These particles often undergo surface recrystallisation into single crystalline polyhedral shell as demonstrated in several examples. Nanocrystalites of zeolite analcime aggregated with ethylamine into large spherical particles, which underwent surface recrystallisation into icositetrahedral single crystal shells.^32^ Spherical aggregates of inorganic precursors for zeolite LTA and biopolymer chitosan developed cubic single-crystal shells.^33^ Aggregates of nanocrystallites of perovskite-type BaTiO_3_ with polyethylene glycol led to dodecahedral crystalline shell.^34^ Spherical aggregates of nanocrystallites of Cu and PVP underwent surface recrystallisation into pseudo-icosahedral shells.^18^ This surface recrystallisation of polycrystalline spheres seems to be quite common.^35^ However, such a surface recrystallisation process was never found in any type of spherulites. The size of the unreduced Cu_2_O crystallites maintains at nanoscale even the reaction time was lengthened to 96 h.

## Conclusions

The formation mechanism of Cu_2_O/PVP composite spherulites has been discussed in detail. PVP shows its multifunction, reducing Cu^2+^ to Cu^+^ as a reducing agent and offering confined space for growth of nanocrystallites of Cu_2_O, but restricting further growth. The soft matter property of PVP allows the embedded nanocrystallites to orient themselves. PVP is also the main component in the cores of the composite spherulites, generating a negative charge centre. The interaction of this central electric field and the Cu_2_O nanocrystallites as dipoles is the driving force for the radially directed self-orientation of the nanocrystallites. This special microstructure leads to a negatively charged surface of spherulites and a high stability of nanocrystallites without further surface recrystallisation into single crystal polyhedral shells. The present work throws light on the investigations of spherulites, polymer assisted crystal growth and the impact of dipolar field in nanocrystallites.

## Author contributions

W. H. Sun is the principal researcher who carried out the experiments and data analysis. He also prepared the draft of the manuscript. W. Z. Zhou supervised this work. He also contributed to the discussions of the experimental results and preparation of the manuscript.

## Conflicts of interest

There are no conflicts to declare.

## Supplementary Material

RA-012-D2RA03302J-s001

## References

[cit1] Shtukenberg A. G., Punin Y. O., Gunn E., Kahr B. (2012). Chem. Rev..

[cit2] Talbot W. H. F. (1837). Philos. Trans. R. Soc. London.

[cit3] Wu S. T., Chiang C.-Y., Zhou W. Z. (2017). Crystals.

[cit4] Sun C.-Y., Gránásy L., Stifler C. A., Zaquin T., Chopdekar R. V., Tamura N., Weaver J. C., Zhang J. A. Y., Goffredo S., Falini G., Marcus M. A., Pusztai T., Schoeppler V., Mass T., Gilbert P. U. P. A. (2021). Acta Biomater..

[cit5] Wu S. T., Blake J. I., Guo L., Zhou W. Z. (2020). Cryst. Growth Des..

[cit6] Connolly B. M., Greer H. F., Zhou W. Z. (2018). Cryst. Growth Des..

[cit7] Greer H. F., Liu M.-H., Mou C.-Y., Zhou W. Z. (2016). CrystEngComm.

[cit8] Cho K.-S., Talapin D. V., Gaschler W., Murray C. B. (2005). J. Am. Chem. Soc..

[cit9] Tang Z. Y., Kotov N. A., Giersig M. (2002). Science.

[cit10] Zhang X., Zhang Z., Glotzer S. C. (2007). J. Phys. Chem. C.

[cit11] Vennum W. R., Eberlein G. D. (1977). J. Res. U.S. Geol. Surv..

[cit12] Studivan M. S., Milstein G., Voss J. D. (2019). PLOS One.

[cit13] Liu M.-H., Tseng Y.-H., Greer H. F., Zhou W. Z., Mou C.-Y. (2012). Chem. – Eur. J..

[cit14] Haque N., Cochrane R. F., Mullis A. M. (2017). Crystals.

[cit15] Woo E. M., Lugito G., Yang C.-E., Chang S.-M. (2017). Crystals.

[cit16] Feng L., Wang K.-Y., Yan T.-H., Zhou H.-C. (2020). Chem.

[cit17] Claes H., Miranda T., Falcão T. C., Soete J., Mohammadi Z., Zieger L., Erthal M. M., Aguillar J., Schmatz J., Busch A., Swennen R. (2021). Mar. Pet. Geol..

[cit18] Sun W. H., Zhou W. Z. (2022). Cryst. Growth Des..

[cit19] Jung H., Lee S. Y., Lee C. W., Cho M. K., Won D. H., Kim C., Oh H.-S., Min B. K., Hwang Y. J. (2019). J. Am. Chem. Soc..

[cit20] Abhilash M. R., Akshatha G., Srikantaswamy S. (2019). RSC Adv..

[cit21] Toe C. Y., Zheng Z., Wu H., Scott J., Amal R., Ng Y. H. (2018). Angew. Chem., Int. Ed..

[cit22] Gou L., Murphy C. J. (2003). Nano Lett..

[cit23] Xue J., Liang W., Liu X., Shen Q., Xu B. (2012). CrystEngComm.

[cit24] Sun S. (2015). Nanoscale.

[cit25] Guo R., Chang J., Li H., He J., Pan P., Yang Z., Wei J. (2022). J. Alloys Compd..

[cit26] Zhu P., Ma Y., Wang Y., Yang Y., Qian G. (2020). J. Mater. Cycles Waste Manage..

[cit27] Song Y.-J., Wang M., Zhang X.-Y., Wu J.-Y., Zhang T. (2014). Nanoscale Res. Lett..

[cit28] Hao C., Zhao Y., Wang D., Lai G. (2012). J. Appl. Polym. Sci..

[cit29] Firmansyah D. A., Kim T., Kim S., Sullivan K., Zachariah M. R., Lee D. (2009). Langmuir.

[cit30] Penn R. L., BanPeld J. F. (1998). Science.

[cit31] Li J.-M. (2017). CrystEngComm.

[cit32] Chen X. Y., Qiao M. H., Xie S. H., Fan K. N., Zhou W. Z., He H. Y. (2007). J. Am. Chem. Soc..

[cit33] Greer H., Wheatley P. S., Ashbrook S. E., Morris R. E., Zhou W. Z. (2009). J. Am. Chem. Soc..

[cit34] Zhan H., Yang X., Wang C., Chen J., Wen Y., Liang C., Greer H. F., Wu M., Zhou W. Z. (2012). Cryst. Growth Des..

[cit35] Zhou W. Z. (2019). Crystals.

