# Supplementary material for: Growth mechanism and microstructures of Cu2O/PVP spherulites
Source: RSC Adv. 2022 Jul 11;12(31):20022–8. doi: 10.1039/d2ra03302j (PMC9272154; doi:10.1039/d2ra03302j)
Supplement: RA-012-D2RA03302J-s001 [file RA-012-D2RA03302J-s001.pdf]

Electronic Supplementary Material (ESI) for RSC Advances.
This journal is © The Royal Society of Chemistry 2022

Electronic Supplementary Information

Growth Mechanism and Microstructures of Cu_2O/PVP Spherulites

Weihao Sun, Wuzong Zhou

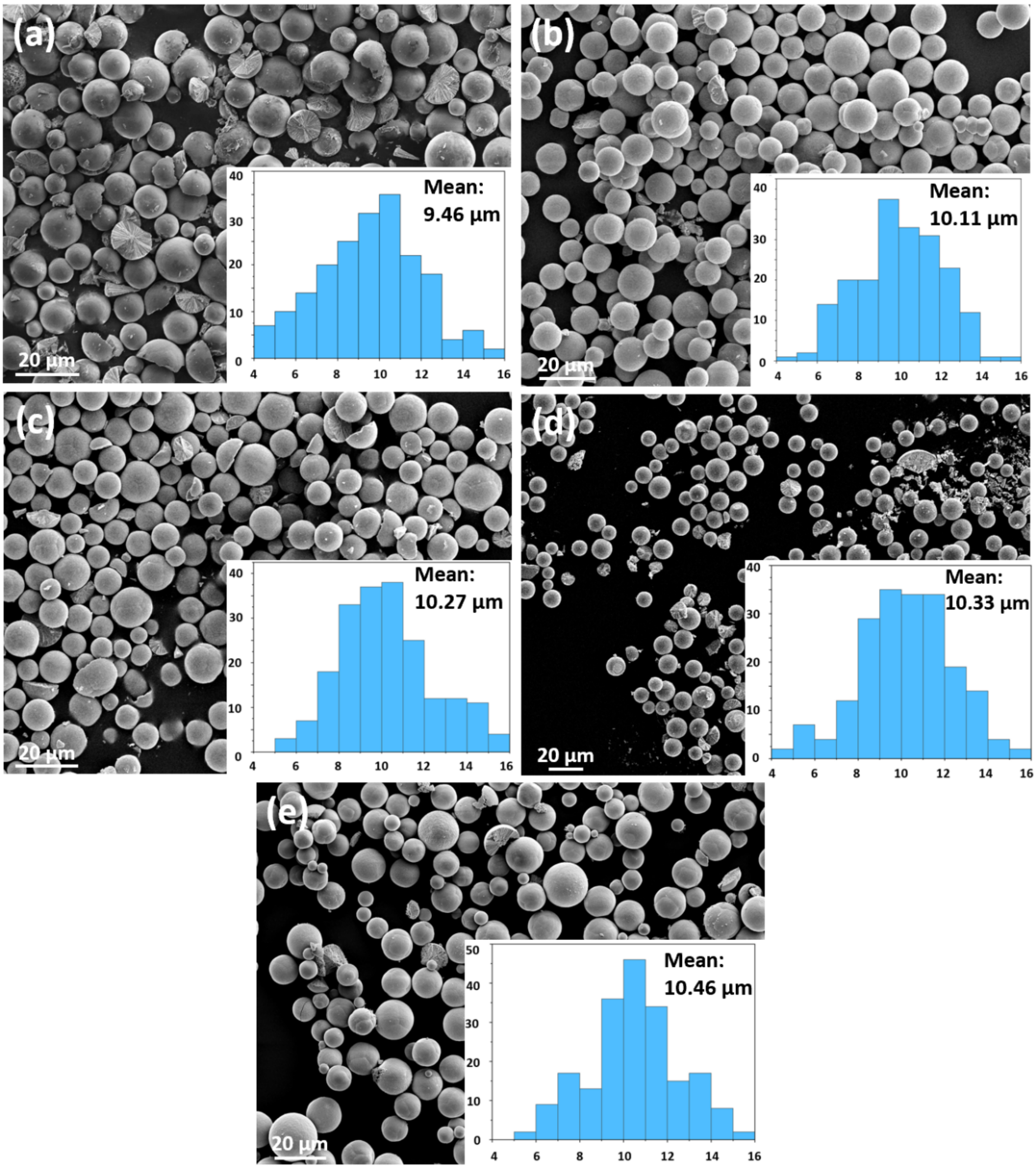

Fig. S1 SEM images of the Cu_2O/PVP spherulites from the samples with reaction times of (a) 1.5, (b) 3, (c) 6, (d) 12 and (e) 96 h. The insets are the corresponding size distribution diagrams of the spherulites. 200 particles for each sample were randomly selected for the measurement.

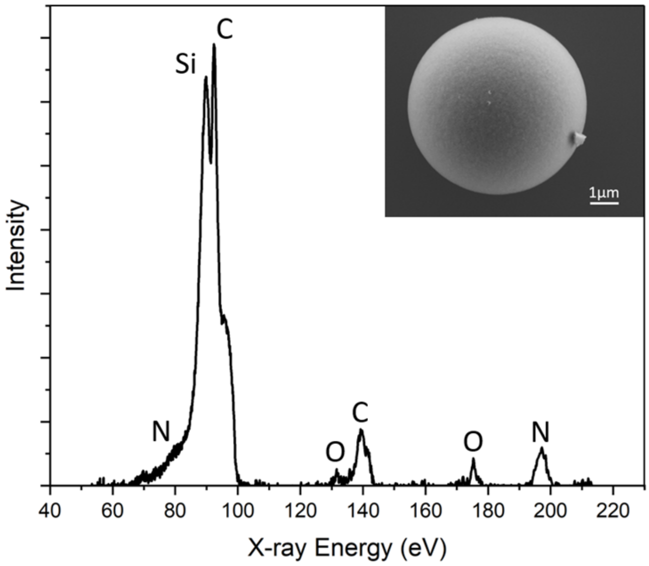

Fig. S2 SXES spectrum of a Cu_2O/PVP spherulite. The Si peak is from the Si substrate. Inset is the corresponding SEM image of the spherulite.

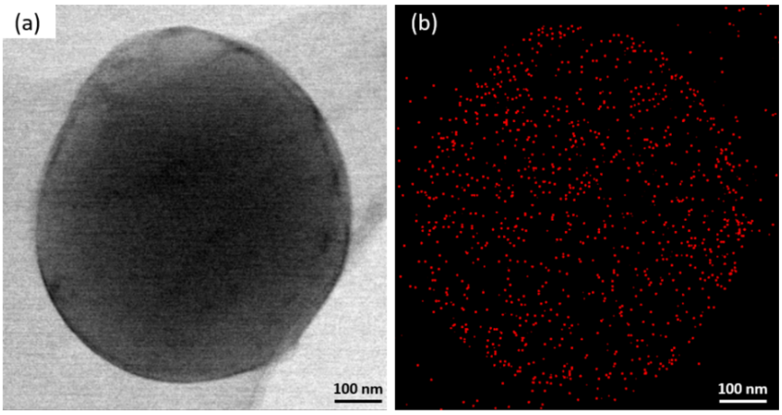

Fig. S3 (a) Bright field TEM image of a submicron sphere from 15 min sample. (b) The corresponding EDX elemental mapping of Cu.

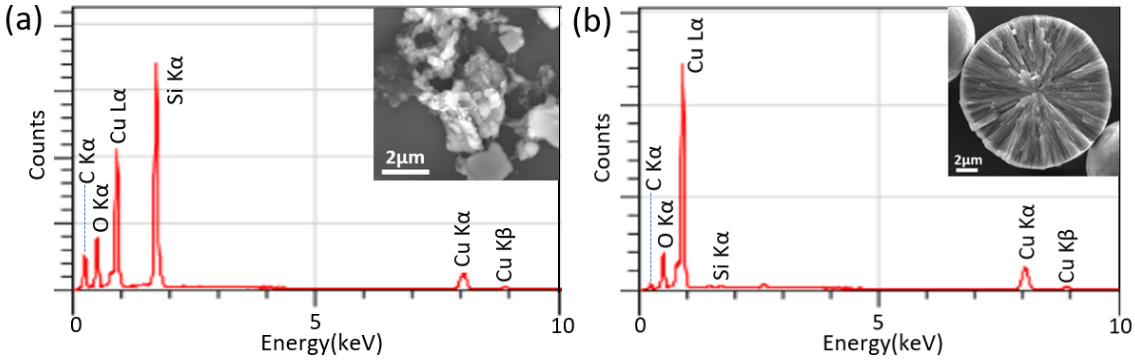

Fig. S4 EDX spectra of (a) the residue in 15 min sample and (b) a spherulite in 1.5 h sample. Insets are the corresponding SEM images of the particles where the spectra were collected.

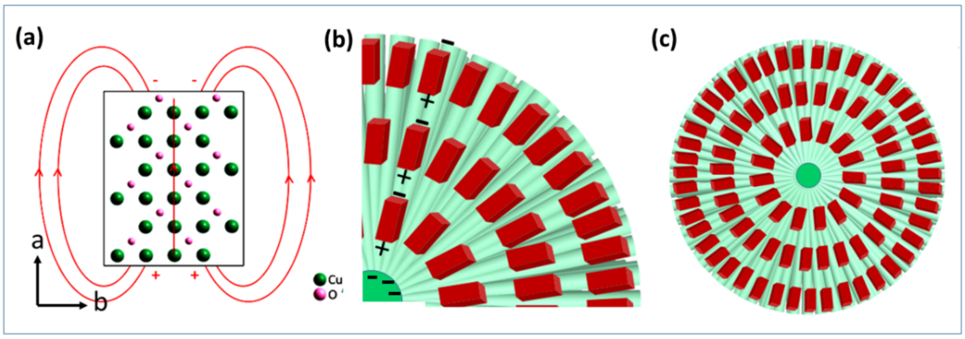

Fig. S5 (a) Structural model of Cu_2O nanocrystal, generating a dipolar field along the [100] axis. (b) Schematic drawing showing the oriented Cu_2O nanocrystllites and corresponding dipole force towards the core. (c) Schematic drawing showing the radial feature of cross section. Red bricks represent Cu_2O nanocrystallites, green substrate represents PVP.

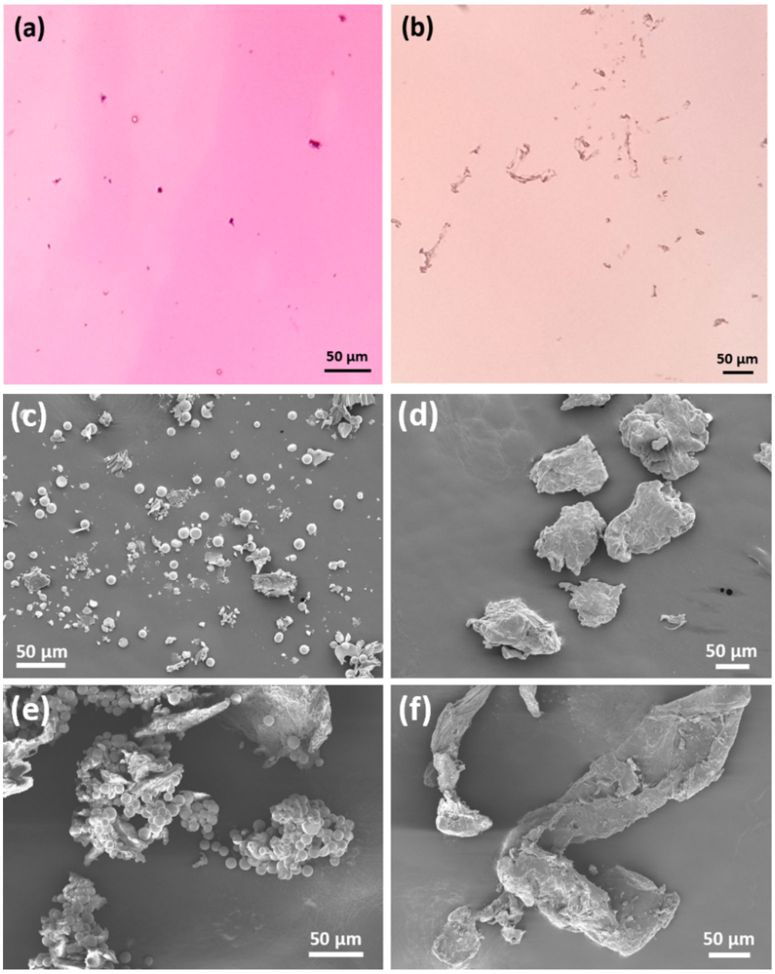

Fig. S6 Optical microscopy images of pure PVP with (a) Safranin T and (b) Congo Red. No obvious interaction happened between PVP and both dyes. SEM images of (c) separated Cu_2O/PVP spherulites from 3 h sample and the negatively charged surfactant, sodium alginate, (d) pure sodium alginate, (e) agglomerated Cu_2O/PVP spherulites on the surface of positively charged surfactant chitosan, (f) pure chitosan.

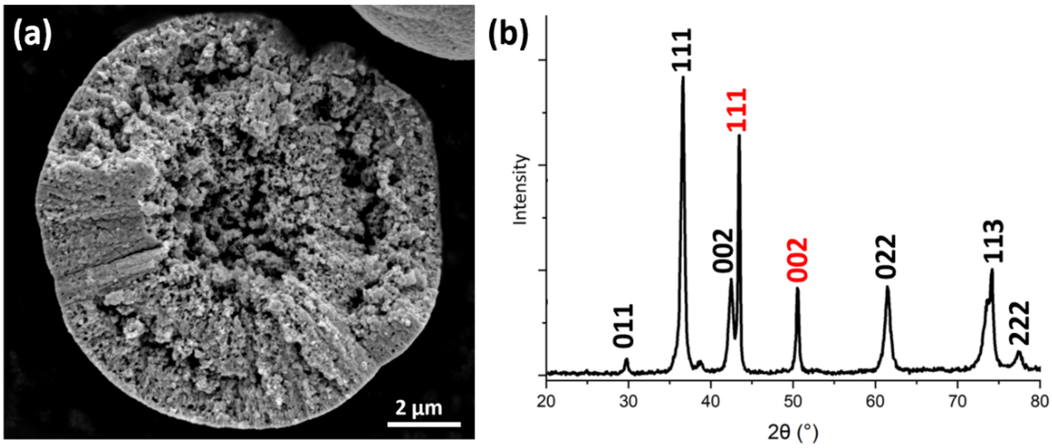

Fig. S7 (a) SEM image of a Cu/PVP spherical particle from 96 h sample. The structure is still spherical while the radial interior with needle-like submicron rods no longer exists. (b) XRD pattern of 96 h sample. Indices marked in black are from cubic Cu_2O while indices marked bright red are from cubic Cu.
